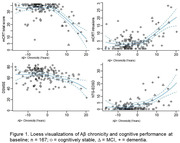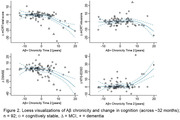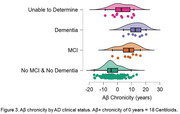# Longitudinal changes in cognition in relation to PET biomarkers

**DOI:** 10.1002/alz.092028

**Published:** 2025-01-09

**Authors:** Sigan L Hartley

**Affiliations:** ^1^ University of Wisconsin‐Madison, Madison, WI USA

## Abstract

**Background:**

Adults with Down syndrome (DS) begin accumulating extracellular brain amyloid‐beta (Aβ) plaques in the decade or two preceding symptomatic Alzheimer’s disease (AD). The present study examined the time course of cognitive impairments cross‐sectionally and across two time points (spaced 32 months) in relation to Aβ chronicity (i.e., estimated years to or from reaching Aβ positivity [Aβ+], defined as 18 Centiloids.

**Methods:**

Analyses included 167 participants with DS (M age = 38.91, SD = 8.41) in the Alzheimer Biomarker Consortium – Down Syndrome (ABC‐DS). Participants completed baseline PET scans using [11C]PiB or [18F]florbetapir and were administered the modified Cued Recall Test (mCRT) and Down Syndrome Mental Status Examination (DSMSE). A study partner completed the National Task Group‐Early Detection Screen for Dementia (NTG‐EDSD). A clinical case consensus approach, blind to neuroimaging data, was used to classify participants as cognitively stable, mild cognitive impairment (MCI), dementia, or unable to determine. Methods were repeated 32 months later. A sampled iterative local approximation algorithm modeled Aβ trajectories longitudinally and assigned each participant a chronicity value, representing timeline to Aβ+ (i.e., Aβ+ = 18 Centiloids) or post Aβ+ (Zammit et al., 2023).

**Results:**

Mixed model Linear regressions showed significant quadradic associations between Aβ chronicity and cognitive performance, both at baseline (Figure 1) and across the 32 months (Figure 2). Using broken stick regression models, declines in mCRT scores began 3.41 years following Aβ+ in longitudinal models, with a decline of 1.35 (95% confidence interval ‐1.85 to ‐0.89) points per year. On average, participants with MCI were estimated to be 7.40 (SD = 6.68) years post Aβ+ and those with dementia were 12.72 (SD = 5.61) years post Aβ+ 9 (Figure 3).

**Conclusion:**

Within‐person memory declines on the mCRT begin at 3 to 4 years following Aβ+ (i.e. Centiloids Aβ+) in DS. After this change point, declines in mCRT were estimated at 1.35 points per year. On average, adults with DS with MCI were 7.4 years post Aβ+ and those with dementia were 12.7 years post Aβ+. Characterizing the timeline of Aβ+ relative to AD symptomology in natural history studies offers estimates for guiding clinical AD trials.